# Investigating the Impact of Gadolinium-Based Contrast Agents on the Corrected QT Interval

**DOI:** 10.7759/cureus.9668

**Published:** 2020-08-11

**Authors:** Kyle L Gress, Tyler Gallo, Ivan Urits, Xue Geng, Omar Viswanath, Alan D Kaye, Raymond L Woosley

**Affiliations:** 1 Department of Pharmacology, Georgetown University School of Medicine, Washington, DC, USA; 2 Division of Clinical Data Analytics, University of Arizona College of Medicine, Phoenix, USA; 3 Department of Anesthesia, Critical Care and Pain Medicine, Beth Israel Deaconess Medical Center, Harvard Medical School, Boston, USA; 4 Department of Biostatistics, Georgetown University, Washington, DC, USA; 5 Pain Management, Valley Pain Consultants - Envision Physician Services, Phoenix, USA; 6 Department of Anesthesiology, Louisiana State University Health Sciences Center, Shreveport, USA; 7 Department of Medicine, University of Arizona College of Medicine, Phoenix, USA

**Keywords:** gadolinium-based contrast agent, drug-induced qtc prolongation, electrocardiogram, pharmacogenetics, arrhythmia

## Abstract

Introduction

The manufacturing labels for all currently marketed gadolinium-based MRI contrast agents describe adverse cardiac events reported during post-market use. The goal of this study was to determine prolongation of the rate-corrected QT interval occurs in the immediate setting after gadolinium-based MRI contrast agent injection.

Methods

This study enrolled adults scheduled to have a gadolinium-based MRI contrast agent injection as part of a diagnostic MRI. A single-lead electrocardiogram was recorded using the AliveCor Kardia® ECG (Mountain View, CA) device before and after injection. The rate-corrected QT interval was subsequently measured by two independent investigators. The QT interval was corrected for rate using the two most common formulas, originally cited by Bazett and Fridericia. These rate-corrected QT intervals from before and after gadolinium-based MRI contrast agent injection were compared using the Wilcoxon signed-rank test paired analysis.

Results

A total of 24 consenting adults had electrocardiogram that were free of motion artifact. The mean age of the final patient cohort was 59.4 years. There was an equal split of 12 men and 12 women. The mean pre-injection, rate-corrected QT interval, corrected using Bazett’s formula, was 395 msec. The mean post-injection, rate-corrected QT interval, corrected using Bazett’s formula, was 396 msec. The corrections using Fridericia's formula were 384 and 381 msec, respectively. There was no statistically significant change in Bazett-corrected QT interval (QTc-B) when pre-injection and post-injection values were directly compared.

Discussion

The results of the present investigation support the conclusion that gadolinium-based MRI contrast agents do not commonly affect rate-corrected QT interval in routine clinical use. While the frequency of rate-corrected QT interval prolongation might be overstated, the severity of adverse events is definitively not. A role for concomitant rate-corrected QT interval-prolonging drugs or unidentified rare factors such as genetic predisposition cannot be ruled out. The limitations of this study include its relatively small size and the implementation of a single-lead electrocardiogram to measure rate-corrected QT interval.

Conclusion

The present investigation revealed that significant rate-corrected QT interval prolongation, while previously reported in as many as 55% of patients after gadolinium-based MRI contrast agent injection, is not a common occurrence in the routine clinical setting.

## Introduction

Chelated gadolinium has been used clinically since 1988 as a contrast dye in MRI [1]. Its paramagnetic nature allows for improved fluid visualization related to enhancement of nuclear relaxation rates [1,2]. The manufacturing labels for each of the nine currently marketed gadolinium-based MRI contrast agents (GCAs) describe adverse cardiac events reported during either safety trials or post-market use. The labels for four currently marketed GCAs mention cardiac arrest and the label for one GCA, gadobenate (MultiHance®), cites commonly occurring rate-corrected QT interval (QTc) prolongation on electrocardiogram (ECG) [3]. QTc prolongation is a well-known clinical biomarker associated with potentially lethal ventricular arrhythmias, torsades de pointes, and sudden death [4]. The MultiHance label cites safety data from a 24-hour, continuous monitoring, crossover study, with a total of 47 patients: QTc changes of at least 30 msec were observed in 26 individuals after gadolinium injection, while only 11 individuals exhibiting these changes with a placebo [3]. According to the United States Food and Drug Administration Adverse Events Reporting System (FAERS) for MultiHance, there have been 73 reports of cardiac arrest and 4 reports of arrhythmia since 2005. Gadoteridol (ProHance®) does not specifically list potential for QTc prolongation on its label but has been cited in 30 reports of cardiac arrest and nine cases of arrhythmia. While the authors are not aware of laboratory research that evaluates the potential for these agents to prolong QTc interval or to have arrhythmogenic effects on cardiac cells, effects of gadolinium on heart tissue have been described, raising the possibility of adverse cardiac events [4]. Furthermore, the current literature also draws connections through case studies to a possible rare side effect of spurious hypocalcemia [5]. The mechanism for this is unclear despite the known connection between hypocalcemia and prolongation of the QTc interval and the unknown effects of established accumulation of gadolinium agents in multiple tissues. Therefore, in the present investigation, possible connections between GCAs and QTc prolongation were evaluated. The goal of this study was to determine whether QTc prolongation would be observed in a routine clinical setting at a rate described in pre-market safety results.

## Materials and methods

To test the hypothesis that GCAs prolong the QTc interval, the present investigation involved a real-world study to measure QTc interval before and after GCA administration for MRI enhancement in the community setting. After obtaining Institutional Review Board (IRB) approval from the University of Arizona IRB, all adult males and females scheduled to undergo MRI with contrast between June and August of 2018 at multiple community hospitals in rural areas in the Midwest region of the United States were invited to participate in the study. Three of the hospitals were serviced by a mobile MRI unit on the back of a semi-truck, while two had their own inhouse MRI suite. For consenting adults, a single-lead ECG was recorded using the AliveCor Kardia® (Mountain View, CA) ECG device before injection of a GCA and after completion of the MRI. This device is approved by the United States Food and Drug Administration (US FDA) for detecting atrial fibrillation and considered comparable to a 12-lead ECG for measuring QTc in healthy volunteers and in inpatients receiving antiarrhythmics known to increase QTc [6].

When patients arrived for diagnostic MRI, they were informed of the study and invited to participate. Consenting adults were surveyed to provide ethnic information, age, sex, current medications, medical history, and reason for undergoing MRI. All included patients agreed to two 30-second ECGs prior to their MRI and two after they received a GCA injection and finished their imaging. The injection for all four gadolinium formulations (ProHance, MultiHance, OptiMARK®, and Omniscan®) was administered as 0.2 milliliter per kilogram as recommended. ECGs were recorded and immediately read by one of two independent physicians to ensure that incidental findings such as an arrhythmia or unknown prolonged QTc intervals were not present. Prolonged QTc was defined with a cutoff of >450 msec for men and >460 msec for women and severely prolonged QTc was defined as >500 msec [7,8]. One patient had a known prolonged QTc. In another case, a patient was referred to their primary physician for evaluation of atrial fibrillation.

Any patient over the age of 18 years scheduled to receive a MRI with contrast was eligible to be enrolled. Exclusion criteria included any patient who had an aborted MRI or whose data were unusable due to motion artifact. All ECG tracings were de-identified and analyzed blindly in randomized order. QT intervals were corrected by rate using the two most common formulas. The Bazett-corrected QT interval (QTc-B) formula uses an inverse-square root function, while the Fridericia-corrected QT (QTc-F) interval formula uses an inverse-cube root function [8,9]. These QTc intervals, QTc-B and QTc-F, were measured by two independent analysts, and all discrepant values were adjudicated to a consensus value by a senior clinician. Using the tangent method, the average QTc-B and QTc-F intervals for three artifact-free cardiac cycles were measured [9].

Pre- and post-injection QTc-B and QTc-F values before and after MRI were compared using the Wilcoxon signed-rank test. All QTc intervals were summarized by median and interquartile range (IQR). The normality of QTc interval data was checked by the Shapiro-Wilk test. Intraclass correlation (ICC) was employed to evaluate reliability for QTc measurements between the two independent analysts at each time point. ICC estimates and their 95% confidence intervals were calculated based on an average unit, consistency type, and two-way model. Based on the 95% confidence interval of the ICC estimate, values less than 0.5, between 0.5 and 0.75, between 0.75 and 0.9, and greater than 0.90 are indicative of poor, moderate, good, and excellent reliability, respectively [10]. All tests were two-sided with a significance level of 0.05 applied. Analyses were performed using the statistical software RStudio.

## Results

Over the course of two months, 32 patients aged 21-93 years were enrolled. Complete data were available for 27 individuals. Five subjects were unable to participate in the MRI related to comorbidities (e.g., claustrophobia, obesity, poor renal function, orthopedic) and the ECGs for three patients could not be interpreted due to excessive motion artifact caused by tremor or chronic arthritis. Each patient received an injection of one of the following GCAs according to manufacturer's instructions: gadoteridol (ProHance), gadobenate (MultiHance), gadoversetamide (OptiMARK), gadodiamide (Omniscan).

Analytically acceptable ECG tracings were obtained for 12 women and 12 men ages 21 to 93 years (mean 59). All patients were Caucasian. Of the 24 patients, 11 had a previous cardiovascular diagnosis; one patient presented with prolonged QTc. Patient demographics are described in Table 1. 

**Table 1:** Patient demographics and cardiac comorbidities of 24 patients. SD, standard deviation

Characteristic	No. (%)
Mean age ± SD, years	59.4 ± 17.7
Sex	
Male	12 (50%)
Female	12 (50%)
Cardiac comorbidities	11 (45%)
Hypertension	8 (33%)
Atrial fibrillation	1 (4%)
Valvular abnormality	3 (13%)
Premature ventricular contraction	1 (4%)
Myocardial infarction	3 (13%)
Congestive heart failure	1 (4%)
Reason for MRI	
Brain	15 (63%)
Cervical spine	3 (13%)
Lumbar spine	2 (8%)
Other	4 (17%)

**Figure 1:**
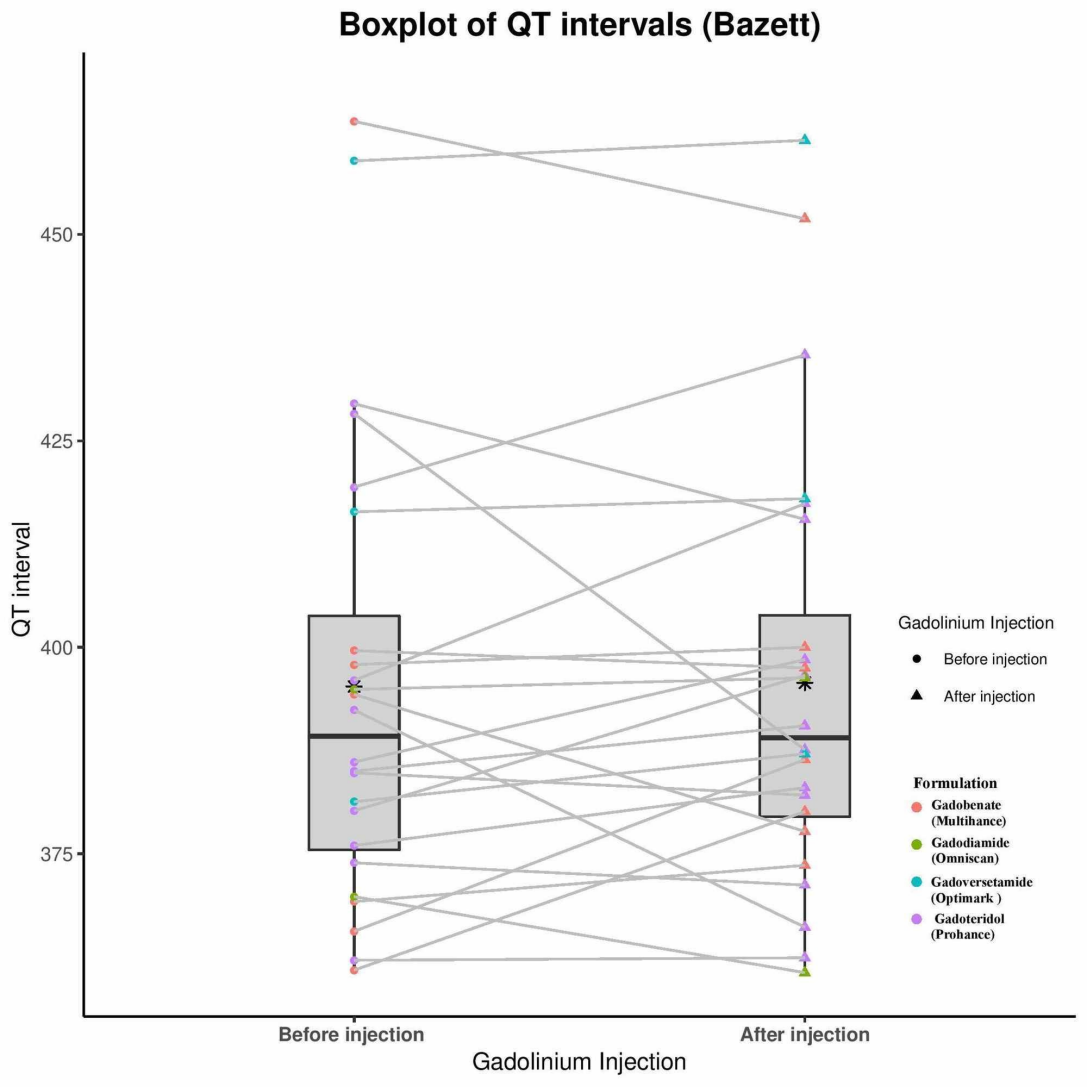
Bazett-corrected QTc intervals. Boxplot showing the minimum, first quantile, median, mean, third quartile, and maximum of the pre-injection and post-injection groups corrected using the Bazett correction QT (QTc-B) values.

**Figure 2:**
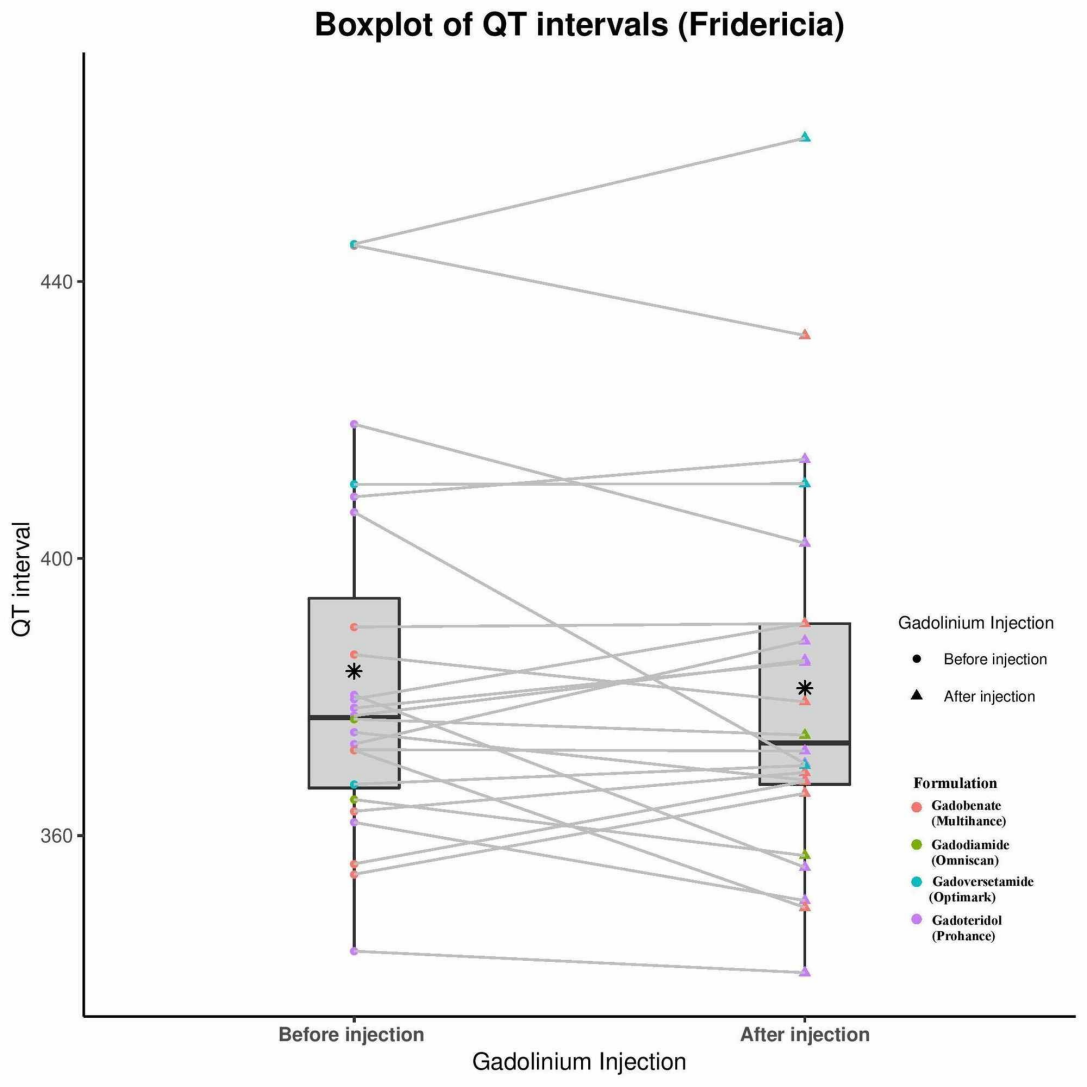
Fridericia-corrected QTc intervals. Boxplot showing the minimum, first quantile, median, mean, third quartile, and maximum of the pre-injection and post-injection groups corrected using the Fridericia correction QT (QTc-F) values.

Statistical analysis was performed that illustrates consistency between the two reviewers measured data. An ICC value greater than 0.9 was considered indicative of excellent reliability, and in this case the values for all four data sets, pre- and post-QT Bazett (QTc-B) and Fridericia (QTc-F) were greater than or equal to 0.970, affirming excellent reliability. Table 2 illustrates these results as well as the minimum, maximum, median, mean, and first and third quartile data for both the pre- and post-injection QTc calculated by both heart rate corrections. The mean pre-injection QTc-B was 395 msec and mean post-injection QTc-B was 396 msec. There was no statistically significant difference by Wilcoxon signed-rank test (p = 0.549). Analysis of the QTc-F data set gave similar results (p = 0.603). Further, there were no subjects that experienced a QTc increase of >30 msec. Secondary analyses for overall increase/decrease in QTc-B, prior cardiac diagnosis, brand of GCA given, age, sex, or hospital did not identify any trends that would suggest that a subset of the population responded differently from the mean. This is illustrated in Figure 1, a box-plot showing the individual QTc-B data points with the data separated by quartile and the overlapping means and standard deviations. Figure 2 provides similar data for QTc-F.

**Table 2:** Overview of statistical analysis performed for both the Bazett and Fridericia corrections Pre-QTc-B: corrected QT interval using Bazett correction measured before gadolinium injection; Post-QTc-B: corrected QT interval using Bazett correction measured after MRI completion; Pre-QTc-F: corrected QT interval using Fridericia correction measured before gadolinium injection; Post-QTc-F: corrected QT interval using Fridericia correction measured after MRI completion; ICC: intraclass correlation

Measurement	Pre-QTc-B	Post-QTc-B	Pre-QTc-F	Post-QTc-F
ICC for QT	0.974	0.970	0.977	0.981
95% confidence interval	(0.939, 0.989)	(0.932, 0.987)	(0.946, 0.990)	(0.956, 0.992)
Minimum (msec)	361	361	343	340
First quantile (msec)	375	380	367	367
Median (msec)	389	389	377	373
Third quantile (msec)	404	404	394	391
Maximum (msec)	464	461	445	461
Standard deviation (msec)	28.1	26.3	26.5	27.6
Mean (msec)	395	396	384	381
P-value	0.549		0.603	

## Discussion

The results of the present investigation support the conclusion that GCAs do not commonly affect QTc in routine clinical use. Excessive QTc prolongation, initially reported in up to 55% of patients after GCA injection, does not appear to be a common occurrence in the acute, routine clinical setting. While the frequency of QTc prolongation might be overstated, the severity of adverse events is definitively not. A role for concomitant QTc-prolonging drugs or unidentified rare factors such as genetic predisposition cannot be ruled out. However, as ECGs become more ubiquitous, pre-injection risk stratification might play a role.

The limitations of this study include its relatively small size and the implementation of a single-lead ECG to measure QTc. While the authors acknowledge that this study is vulnerable to a type II (β) error, even with a small sample size, the lack of a common trend in the data suggests that GCA do not routine cause QTc prolongation at the reported frequency of 55% [3]. A larger sample size and administration of a single type of GCA agent would have greater power to detect small changes. One might critique the use of a single-lead ECG device as inferior to a hospital grade 12-lead ECG; however, while using a 12-lead ECG may facilitate the finding of subtle changes in different leads, QTc measurements using the AliveCor single-lead, mobile device have been shown previously to compare equivocally with a standard 12-lead ECG and agree within 5 msec [6]. Furthermore, the study could have been confounded due to an individual's usage of QTc prolonging drugs; more than 100 drugs currently on the market have possible QTc prolongation as a known side effect. The authors considered this an acute study looking at immediate response of the subject's QTc to GCA injection and assumed that individuals with chronic, drug-induced QTc prolongation would be identified on the initial pre-injection screening, before injection. Since the patients enrolled in the study were all Caucasian and from a relatively confined geographic area in northeast Kansas and southern Nebraska, there is a possibility that GCAs could affect individuals from other ethnicities or regions differently. Nevertheless, when looking at this real-world experience, the results suggest an absence of large or frequent changes in QTc as previously published, improving our current understanding of the overall safety profiles of these GCAs.

## Conclusions

The manufacturing labels for all nine of the currently marketed gadolinium-based MRI contrast agents describe adverse cardiac events, including QTc prolongation, as known and common side effects. The authors recognize laboratory research showing the potential for these agents to prolong to have effects on cardiac tissue. Consenting adults were subjected to sixty seconds of continuous ECG monitoring before and after receiving a GCA injection. These ECGs were then analyzed for QTc changes. Although a small study, the results suggest that there are not frequent prolongations in QTc as previously published. These data improve upon the current understanding of the overall safety profiles of these GCAs.
